# Supplementary material for: Perioperative management of angiotensin-converting enzyme inhibitors and/or angiotensin receptor blockers: a survey of perioperative medicine practitioners
Source: PeerJ. 2018 Jun 29;6:e5061. doi: 10.7717/peerj.5061 (PMC6055831; doi:10.7717/peerj.5061)
Supplement: Appendix S1 [file peerj-06-5061-s001.docx]

| SURVEY CHECKLIST  Literature review: p 1-3. |
| --- |
|  |
| Ethics approval: p.3 |
| Sample Preferred: p.4 |
|  |
| Sample size: p.4 |
|  |
| Design: p.3-4 |
|  |
| Pilot:p.4 |
|  |
| Maximize response : p.4 |
|  |
| Response rate: p.6 |
|  |
| Statistics: p.5 |
|  |
| Comparison: p.6-7 |
|  |
| Confidence intervals: throughout |
|  |
| Conclusion: p.9 |
|  |
| Limitations: p.9 |
|  |
| Survey access: p.4 |


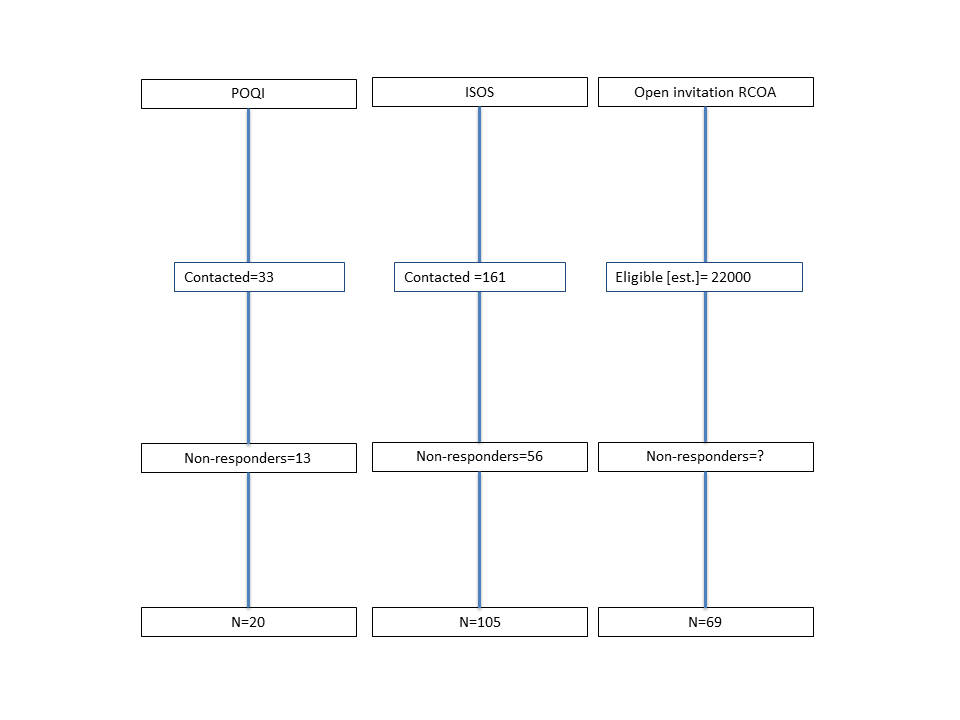
**Correspondents response rate**
