# Supplementary material for: Perioperative management of angiotensin-converting enzyme inhibitors and/or angiotensin receptor blockers: a survey of perioperative medicine practitioners
Source: PeerJ. 2018 Jun 29;6:e5061. doi: 10.7717/peerj.5061 (PMC6055831; doi:10.7717/peerj.5061)
Supplement: Appendix S2 [file peerj-06-5061-s002.docx]

**Appendix 2-Questionnaire**

**A survey launched in conjunction with a randomized controlled trial [ISRCTN17251494]**

**exploring stopping or continuing perioperative angiotensin II converting enzyme inhibitors and/ or angiotensin receptor blockers in patients undergoing major noncardiac surgery.**

**Supported by the British Oxygen Company/Royal College of Anaesthetists 2015 award.**

***We would be grateful if you could complete this survey, which takes approximately 5 minutes. Many thanks for considering.*** [g.ackland@qmul.ac.uk](mailto:g.ackland@qmul.ac.uk)

**Question 1**

With regard to ACE inhibitors, what do you advise patients prior to major surgery? (Please select the SINGLE option that is closest to your usual practice)

- Continue ACEi
- Stop 3 days before surgery
- Stop 2 days before surgery
- Stop 1 day before surgery
- Stop on day of surgery
- Stop (duration of stopping dependent on the drug)

**Question 2**

With regard to ACE inhibitors, when would you normally plan to re-start them after major surgery? (Please select the SINGLE option that is closest to your usual practice)

- Within 12 hours after surgery
- 12-24 hours after surgery
- 24 hours after surgery
- 48 hours after surgery

**Question 3**

With regard to Angiotensin Receptor Blockers (e.g. Candesartan/Irbesartan/ Losartan/ Olmesartan/ Telmisartan/ Valsartan) what do you advise patients prior to major surgery? (Please select the SINGLE option that is closest to your usual practice)

- Continue ARB
- Stop 3 days before surgery
- Stop 2 days before surgery
- Stop 1 day before surgery
- Stop on day of surgery
- Stop (duration of stopping dependent on the drug)

**Question 4**

With regard to Angiotensin Receptor Blockers, when would you normally plan to re-start these drugs after major surgery? (Please select one answer)

- Within 12 hours after surgery
- 12-24 hours after surgery
- 24 hours after surgery
- 48 hours after surgery

**Question 5**

Which of these preop cardiovascular medications do you routinely stop prior to major surgery? (You may choose more than one option)

- Aliskerin
- Amiodarone
- Amlodipine
- Atenolol
- Bisoprolol
- Bendroflumethiazide
- Carvediol
- Digoxin
- Diltiazem
- Doxazosin
- Eplerenone
- Felodipine
- Furosemide
- Indapamide
- Isosorbide mononitrate
- Metoprolol
- Spironolactone
- Verapamil
- Other diuretics

**Question 6**

Preop Case Scenario: A 65 year old patient (ASA Grade 3) with an anaerobic threshold <10ml.kg.min-1 has an elevated blood pressure (>160 systolic, >100 diastolic) on the day of their reversal of Hartmann’s procedure. They are NOT on any anti-hypertensive medications. What would you do? (Please select the SINGLE option that is closest to your usual practice)

- Postpone the procedure
- Treat acutely to reduce BP to <160/<100 then proceed
- Proceed, but change the post-operative management plan to involve HDU
- Proceed, but make an in hospital medical referral
- Proceed as planned

**Question 7**

Preop Case Scenario:  A 65 year old patient (ASA Grade 3) with an anaerobic threshold <10ml.kg.min-1 has an elevated blood pressure (>160 systolic, >100 diastolic) on the day of their reversal of Hartmann’s procedure. They are normally on an ACE-i or ARB to control their blood pressure, but this was stopped within the last 48 hours. What would you do? (Please select the SINGLE option that is closest to your usual practice)

- Postpone the procedure
- Treat acutely to reduce BP to <160/<100 then proceed
- Proceed, but change the post-operative management plan to involve HDU
- Proceed, but make an in hospital medical referral
- Proceed as planned

**Question 8**

Preop Case Scenario:  A 65 year old patient (ASA Grade 3) with an anaerobic threshold <10ml.kg.min-1 has an elevated blood pressure (>160 systolic, >100 diastolic) on the day of their reversal of Hartmann’s procedure. They are normally on an ACE-i or ARB to control their blood pressure. This has not been stopped. What would you do? (Please select the SINGLE option that is closest to your usual practice)

- Postpone the procedure
- Treat acutely to reduce BP to <160/<100 then proceed
- Proceed, but change the post-operative management plan to involve HDU
- Proceed, but make an in hospital medical referral
- Proceed as planned

**Question 9**

Postop Case Scenario: A 65 year old patient (ASA Grade 3) is Day 1 post-op following a colonic resection. They usually receive ACE-i or ARB medication for hypertension. They are clinically stable with no signs of sepsis, good urine output, normal blood gases and normal lactate. Their systolic blood pressure is consistently 90-100mmHg. Would you restart their usual ACE-i/ARB therapy? (Please select the SINGLE option that is closest to your usual practice)

- Yes
- No

**Question 10**

Postop Case Scenario: A 65 year old patient (ASA Grade 3) is Day 1 post-op following a colonic resection.  They were on ACE-i/ARB prior to surgery. Although they have no chest pain and have not developed any ECG changes, an elevated troponin level has been recorded. They are pain-free, clinically and haemodynamically stable. Their systolic BP is consistently >170mmHg. Please select the SINGLE option that is closest to your usual practice

- Restart their ACEi of ARB
- Treat acutely to reduce systolic BP to <160mmHg
- Start long-acting anti-hypertensives
- Refer for medical advice
- Continue without intervention but monitor

**Question 11**

Postop Case Scenario: A 65 year old patient (ASA Grade 3) is Day 1 post-op following a colonic resection. They are pain-free, clinically and haemodynamically stable. An elevated plasma troponin level has been noted, but they have no chest pain and have not developed any ECG changes. Their systolic BP is consistently >170mmHg. They were NOT on ACE-I/ARB prior to surgery. Please select the SINGLE option that is closest to your usual practice

- Start an ACEi of ARB
- Treat acutely to reduce systolic BP to <160mmHg
- Start long-acting anti-hypertensives
- Refer for medical advice
- Continue without intervention but monitor

**Question 12**

Any additional comments regarding perioperative management of ACE inhibitors/ angiotensin receptor blockers?

**Question 13**

Please record your clinical background/grade

- Anaesthesia/ICU-consultant/staff grade
- Anaesthesia/ICU-trainee
- Preoperative assessment nurse
- Surgeon-consultant
- Surgeon-trainee
- General medicine-consultant
- General medicine-trainee
- Cardiology-consultant
- Cardiology-trainee
- Gerontology-consultant
- Gerontology-trainee

**Question 14**

Please indicate your location of practice.

- UK
- Other European Country (inc Russia)
- Canada
- USA
- Mexico
- Central America
- South America
- Asia
- Indian subcontinent
- Australasia
- Africa
