# Supplementary material for: Perioperative management of angiotensin-converting enzyme inhibitors and/or angiotensin receptor blockers: a survey of perioperative medicine practitioners
Source: PeerJ. 2018 Jun 29;6:e5061. doi: 10.7717/peerj.5061 (PMC6055831; doi:10.7717/peerj.5061)
Supplement: Appendix S3 — Each centre had one potential correspondent principal investigator, unless stated otherwise. [file peerj-06-5061-s003.docx]

**Appendix 3: UK ISOS investigator centres.** Each centre had one potential correspondent principal investigator, unless stated otherwise.

1. Aintree University Hospital
2. Airedale General Hospital
3. Ashford Hospital
4. Barnet General Hospital (3 PIs)
5. Basildon and Thurrock Hospital
6. Basingstoke North Hampshire Hospital
7. Birmingham Women’s Hospital (2 PIs)
8. Blackpool Victoria Hospital
9. Bradford Teaching Hospital
10. Bristol Royal Infirmary (2 PIs)
11. Bristol Royal Infirmary
12. Bronglais General Hospital
13. Broomfield Hospital
14. Burnley General Hospital
15. Castle Hill Hospital (2 PIs)
16. Charing Cross Hospital
17. Cheltenham General Hospital (2 PIs)
18. Chorley and South Ribble Hospital
19. Colchester General Hospital
20. Darlington Memorial Hospital (2 PIs)
21. Derriford Hospital
22. Dewsbury Hospital
23. Diana Princess of Wales Hospital
24. Doncaster Royal Infirmary
25. Dorset County Hospital (2 PIs)
26. Frenchay Hospital
27. Furness General Hospital
28. Glenfield Hospital
29. Gloucestershire Royal Hospital
30. Great Western Hospital
31. Guy's Hospital (2 PIs)
32. Hammersmith Hospital
33. Harrogate District Hospital (2 PIs)
34. Hexham Hospital
35. Homerton University Hospital
36. Hull Royal Infirmary (2 PIs)
37. Ipswich Hospital
38. James Cook University Hospital (3 PIs)
39. Kettering General Hospital
40. King's College Hospital (3 PIs)
41. King's Mill Hospital
42. Leicester General Hospital
43. Leicester Royal Infirmary
44. Lister Hospital
45. London Chest Hospital
46. Maidstone Hospital
47. Manchester Royal Eye Hospital
48. Manchester Royal Infirmary
49. Medway Maritime Hospital (2 PIs)
50. Musgrove Park Hospital
51. New Cross Hospital (2 PIs)
52. Newham University Hospital
53. Norfolk and Norwich University Hospital (2 PIs)
54. North Devon District Hospital (2 PIs)
55. North Manchester General Hospital
56. North Tyneside General
57. Northampton General Hospital
58. Northern General Hospital
59. Nottingham University Hospitals (2 PIs)
60. Papworth Hospital
61. Pinderfields Hospital (2 PIs)
62. Pontefract Hospital
63. Poole Hospital (2 PIs)
64. Prince Charles Hospital
65. Queen Alexandra Hospital
66. Queen Elizabeth Hospital Birmingham
67. Queen Elizabeth Hospital Kings Lynn
68. Queen Victoria Hospital (2 PIs)
69. Queens Hospital (2 PIs)
70. Robert Jones and Agnes Hunt Orthopaedic Hospital
71. Rotherham Hospital
72. Royal Blackburn Hospital
73. Royal Cornwall Hospital (2 PIs)
74. Royal Derby Hospital
75. Royal Devon and Exeter Hospital
76. Royal Free Hospital
77. Royal Glamorgan Hospital
78. Royal Gwent Hospital (2 PIs)
79. Royal Hallamshire Hospital
80. Royal Hampshire County Hospital
81. Royal Infirmary Edinburgh (2 PIs)
82. Royal Lancaster Infirmary
83. Royal London Hospital
84. Royal Preston Hospital (2 PIs)
85. Royal Surrey County Hospital
86. Royal Sussex County Hospital (2 PIs)
87. Royal United Hospital Bath (2 PIs)
88. Royal Victoria Infirmary
89. Russells Hall Hospital
90. Saint Mary's Hospital
91. Salisbury District Hospital (2 PIs)
92. Sandwell & West Birmingham Hospitals NHS Trust
93. Scarborough Hospital
94. Scunthorpe General Hospital (2 PIs)
95. Southend University Hospital (2 PIs)
96. Southmead Hospital
97. St Mary's Hospital, London (2 PIs)
98. St Peter's Hospital
99. St Thomas' Hospital (2 PIs)
100. Sunderland Royal Hospital (2 PIs)
101. Royal Oldham Hospital
102. Royal Orthopaedic Hospital
103. Torbay Hospital (2 PIs)
104. Trafford Hospitals
105. Tunbridge Wells Hospital
106. University College London
107. University Hospital of North Durham
108. University Hospital of North Tees
109. University Hospital of Wales
110. University Hospital Southampton
111. Wansbeck General Hospital
112. Watford General
113. West Suffolk Hospital
114. Weston General Hospital (2 PIs)
115. Whipps Cross University Hospital
116. Whittington Hospital (3 PIs)
117. Worcestershire Royal Hospital (2 PIs)
118. Yeovil District Hospital
119. York Hospital
