# Supplementary figures and images for: Perioperative management of angiotensin-converting enzyme inhibitors and/or angiotensin receptor blockers: a survey of perioperative medicine practitioners
Source: PeerJ. 2018 Jun 29;6:e5061. doi: 10.7717/peerj.5061 (PMC6055831; doi:10.7717/peerj.5061)

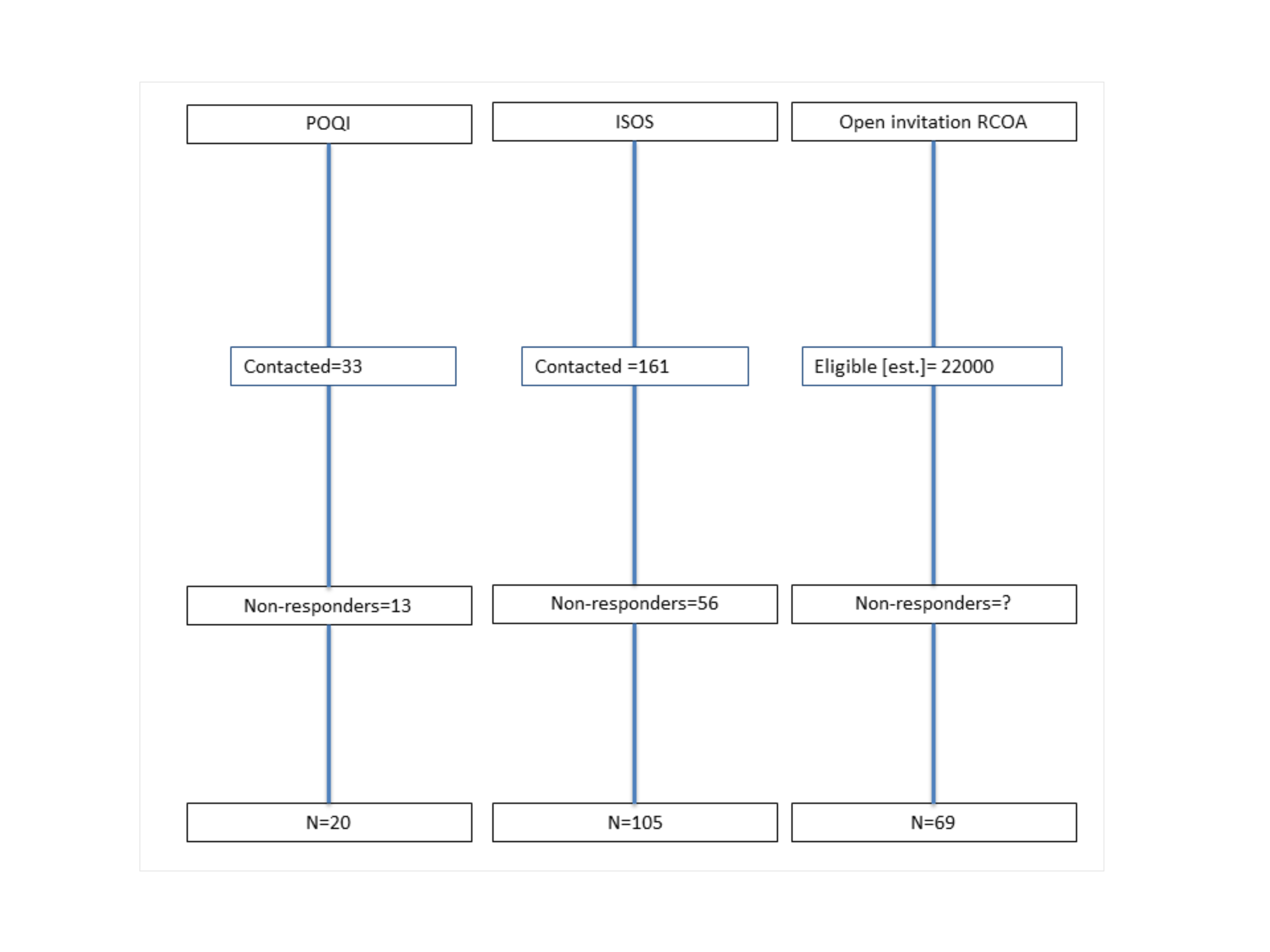

Supplement: Figure S1 [file peerj-06-5061-s005.png]
